# Alphavirus-Based Vaccines

**DOI:** 10.3390/v6062392

**Published:** 2014-06-16

**Authors:** Kenneth Lundstrom

**Affiliations:** PanTherapeutics, Rue des Remparts 4, CH1095 Lutry, Switzerland; E-Mail: lundstromkenneth@gmail.com; Tel.: +41-79-776-6351

**Keywords:** alphaviruses, vaccines, naked RNA, DNA vaccines, recombinant particles, protection against lethal virus challenges, tumor protection, clinical trials

## Abstract

Alphavirus vectors have demonstrated high levels of transient heterologous gene expression both *in vitro* and *in vivo* and, therefore, possess attractive features for vaccine development. The most commonly used delivery vectors are based on three single-stranded encapsulated alphaviruses, namely Semliki Forest virus, Sindbis virus and Venezuelan equine encephalitis virus. Alphavirus vectors have been applied as replication-deficient recombinant viral particles and, more recently, as replication-proficient particles. Moreover, *in vitro* transcribed RNA, as well as layered DNA vectors have been applied for immunization. A large number of highly immunogenic viral structural proteins expressed from alphavirus vectors have elicited strong neutralizing antibody responses in multispecies animal models. Furthermore, immunization studies have demonstrated robust protection against challenges with lethal doses of virus in rodents and primates. Similarly, vaccination with alphavirus vectors expressing tumor antigens resulted in prophylactic protection against challenges with tumor-inducing cancerous cells. As certain alphaviruses, such as Chikungunya virus, have been associated with epidemics in animals and humans, attention has also been paid to the development of vaccines against alphaviruses themselves. Recent progress in alphavirus vector development and vaccine technology has allowed conducting clinical trials in humans.

## 1. Introduction

Alphaviruses are single-stranded RNA viruses with an envelope structure belonging to the family of Togaviridae [1]. Certain alphaviruses have been associated with pathogenicity, resulting in global fever epidemics, such as observed recently for Chikungunya virus [2]. Furthermore, Semliki Forest virus (SFV) [3] and Venezuelan equine encephalitis (VEE) virus [4] have been identified as the causes of an outbreak of febrile illness in Central Africa and an epidemic in horses and humans in South America, respectively. Despite this potential concern, several alphaviruses, including SFV [5], Sindbis virus (SIN) [6] and VEE [7] have been subjected to the engineering of vectors for heterologous gene expression. In these cases, attenuated strains have been employed.

**Figure 1:**
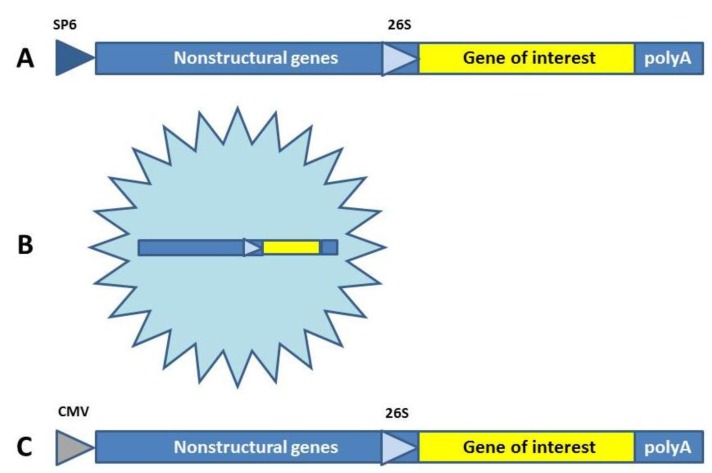
Alphavirus vector systems for vaccine delivery. (**A**) Naked RNA vector *in vitro* transcribed from plasmid DNA. (**B**) Replication-deficient alphavirus particles generated in baby hamster kidney (BHK) cells after co-transfection of *in vitro* transcribed RNA from the expression and helper vectors. (**C**) Layered DNA vector for plasmid immunization. SP6, polymerase promoter; 26S, subgenomic alphavirus promoter; CMV, cytomegalovirus promoter; polyA tail, polyadenylation signal.

Several types of vector systems have been engineered. There are three types of replication-deficient vectors consisting of naked RNA, recombinant particles and layered DNA vectors (Figure 1). The application of naked RNA vectors involves the use of *in vitro* transcribed RNA from an expression vector consisting of the viral nonstructural replicase genes and the foreign gene of interest downstream of the strong subgenomic promoter. The production of recombinant particles requires the co-transfection of *in vitro* transcribed RNA from an expression vector (as described above) and a helper vector supplying the viral structural genes into mammalian cell lines (for example, baby hamster kidney (BHK) cells). The generated particles are capable of one round of infection of a broad range of host cells, but due to the selective packaging of only expression vector RNA, no further virus production occurs. The layered DNA vector system consists of delivery of a DNA vector providing foreign gene expression from a CMV promoter. Furthermore, the engineering of vectors with an additional subgenomic promoter to the full-length genome allows for the generation of replication-proficient particles, which can provide improved delivery and extended gene expression. All alphavirus vectors described take advantage of the extremely efficient RNA replication, resulting in some 200,000 RNA copies from each RNA molecule. The essential question is: which vector system to use? Obviously, replication-proficient particles can provide efficient delivery, but suffer from potential insufficiency related to safety aspects. Although replication-deficient particles provide a higher level of safety, there is still a marginal risk of the generation of replication-proficient particles through non-homologous recombination. To minimize any unwanted recombination events, a split helper vector system with capsid and envelope genes expressed from separate helper vectors has been engineered [8].

So far, alphaviruses have been applied for the expression of a number of topologically different recombinant proteins [9]. Particularly, the use of SFV particles has resulted in high expression levels of integral membrane proteins in various mammalian host cell lines [10], in primary neurons [11] and *in vivo* [12]. For vaccine development, vectors based on SFV, SIN and VEE have been applied as naked RNA, recombinant virus particles and layered DNA vectors [13]. In this context, viral and tumor antigens have been administered in various animal models to elicit neutralizing antibodies and protection against challenges with tumor cells or lethal doses of viruses. Moreover, non-viral pathogens have been subjected to vaccine development. Replicon particles derived from VEE have furthermore demonstrated activity as safe and potent systemic, mucosal and cellular adjuvants when co-administered with antigen [14]. Finally, as alphaviruses have been identified as the cause of viral epidemics in animals and humans, a number of approaches have been initiated for immunization against alphavirus-based infections. In this review, the latest development on alphavirus vectors for vaccine production is summarized.

## 2. Viral Vaccine Approaches

Due to their immunogenic properties, viral structural proteins have been popular targets for alphavirus-based vaccine development [13] (Table 1). In this context, immunization with SFV particles expressing influenza nucleoprotein (NP) elicited a strong immune response in mice [15]. Moreover, VEE-based expression of influenza hemagglutinin (HA) provided protection against challenges with H5N1 virus in chicken [16]. Similarly, SFV particles expressing the HIV envelope [17] and gp41 [18] and VEE particles expressing HIV MA/CA [19] showed humoral and CTL (cytotoxic T-lymphocyte) responses in mice. Furthermore, a VEE particle vaccine expressing the cluster IV H3N2 swine influenza HA gene demonstrated protection against challenges with homologous influenza virus [20]. Likewise, mice and guinea pigs vaccinated with VEE particles expressing Ebola NP [21] and GP [22], respectively, provided protection against challenges with lethal doses of Ebola virus.

**Table 1:** Alphavirus-based vaccine development for viral targets.

Virus	Target	Vector/Delivery	Immunization	Response	Reference
BVDV	E2	VEE/Particles	Calf	BVDV protection	[23]
	NS3 (p80)	SFV/DNA	Mouse	CTL, CMI	[24]
CMV	gB/pp65-1E1	VEE/Particles	Human Phase I	Neutralizing Abs	[25]
CSFV	E2	SFV/DNA	Swine	CSFV protection	[26]
Dengue	PrME, E85	VEE/Particles	Macaque	Dengue protection	[27]
Ebola	NP	VEE/Particles	Mouse	Ebola protection	[21]
	NP, GP	VEE/Particles	Guinea pig	Ebola protection	[22]
	VP24, 30, 35, 30	VEE/Particles	Mouse	Ebola protection	[28]
Hepatitis B	cAg	SIN/DNA	Mouse	Specific Abs	[29]
	sAg	SIN/DNA	Mouse	Specific Abs	[29]
Hepatitis C	cAg	SFV/Particles, DNA	Mouse	CTL	[30]
	NS3	SFV/Particles	Mouse	Cellular	[31]
	nsPs	SFV/Particles	Mouse	CD8^+^ T-cell response	[32]
HeV	Glycoprotein	VEE/Particles	Mouse	Neutralizing Abs	[33]
HIV-1	Env	SFV/Particles	Mouse	Humoral	[17]
	gp41	SFV/Particles	Mouse	Monoclonal Abs	[18]
	MA/CA	VEE/Particles	Mouse	Humoral, CTL	[19]
HPV	16E7	SFV/DNA	Mouse	CTL	[34]
	16E7-VP22	SIN/Particles	Mouse	CD8^+^ T-cell response	[35]
HSV-1	gpB	SIN/Particles	Mouse	HSV protection	[36]
	gpB	SIN/DNA	Mouse	CTL, protection	[37]
IBDV	VP2	SFV/Particles, DNA	Chicken	Specific Abs	[38]
Influenza	HA	SFV/Particles	Mouse	Systemic response	[15]
	HA	VEE/Particles	Chicken	Influenza protection	[16]
	HA	VEE/Particles	Swine	Influenza protection	[20]
	NP	SFV/Particles, RNA	Mouse	Humoral, CTL	[39]
ISAV	HE	SAV/Particles	Salmon	ISAV protection	[40]
JEV	prM-E, NS1-2A	SIN/Particles	Mouse	JEV Abs	[41]
Lassa	N	VEE/Particles	Mouse	Immune response	[42]
LIV	prME	SFV/Particles	Mouse	LIV protection	[43]
	prME, NS1	SFV/Particles	Sheep	LIV protection	[44]
MBGV	GP, NP, VP35	VEE/Particles	Guinea pig	MBGV protection	[45]
	GP, NP	VEE/Particles	Macaque	MBGV protection	[46]
Measles	HA, FUd	SIN/DNA	Mouse	Measles protection	[47]
	HA, FUd	SIN-VEE/Particles	Macaque	Measles protection	[48]
MVE	prME, E	SFV/Particles	Mouse	Neutralizing Abs	[49]
NiV	Glycoproteins	VEE/Particles	Mouse	Neutralizing Abs	[33]
NLV	VLP	VEE/Particles	Mouse	Immune response	[50]
Rabies	G	SIN/DNA	Mouse	Rabies protection	[51]
RSV	F, G	SFV/DNA, RNA	Mouse	RSV protection	[52]
	F, G	SFV/Particles	Mouse	RSV protection	[53]
RVFV	Gn	VEE/Particles	Mouse	RVFV protection	[54]
SARS-CoV	Glycoprotein	VEE/Particles	Mouse	SARS-CoV protection	[55]
SEOV	M, S	SIN/Particles, DNA	Hamster	SEOV protection	[56]
SHIV	Env	SFV/Particles	Macaque	T-cell proliferative response	[57]
SUDV	GP	VEE/Particles	Primate	SUDV protection	[58]
Vaccinia	A33R, B5R	VEE/Particles	Mouse	Vaccinia protection	[59]

Abbreviations: Abs, antibodies; BVDV, bovine viral diarrhea virus; CMI, cell-mediated immune response; CMV, cytomegalovirus; CSFV, classical swine fever virus; CTL, cytotoxic T-lymphocyte activity; HBV, hepatitis B virus; HBC, hepatitis C virus; HE, hemagglutinin-esterase; HeV, Hendra virus; HIV, human immunodeficiency virus; HPV, human papillomavirus; HSV, herpes simplex virus; IBDV, infectious bursal disease virus; ISAV, infectious salmon anemia virus; JEV, Japanese encephalitis virus; LIV, Louping-ill virus; MBGV, Marburg virus; MVE, Murray Valley encephalitis virus; NiV, Nipah virus; NLV, Norwalk-like virus; RSV, respiratory syncytial virus; RVFV, Rift Valley fever virus; SARS‑CoV, severe acute respiratory syndrome corona virus; SAV, salmon anemia virus; SEOV, Seoul virus; SFV, Semliki Forest virus; SHIV, simian-human immunodeficiency virus; SIN, Sindbis virus; SUDV, Sudan virus; VEE, Venezuelan equine encephalitis virus.

Attempts to further improve the immunogenicity of vaccine candidates, the herpes simplex virus type I (HSV-1) VP22 protein was fused to the H5N1 subtype influenza HA [60]. The responses of both interleukin-4 (IL-4) of CD4^+^ T-cells and interferon-gamma (IFNɣ) of CD8^+^ T-cells were observed in vaccinated mice. VEE replicon particles expressing the severe acute respiratory syndrome coronavirus (SARS-CoV) glycoprotein managed to provide protection against challenges with lethal doses of SARS-CoV in vaccinated mice [55]. Furthermore, VEE particles were applied for the expression of glycoproteins from the zoonotic pathogenic Hendra virus (HeV) and Nipah virus (NiV), known to cause fatal infections in both animals and humans [33]. Immunization resulted in enhanced induction of cross-reactive neutralizing antibodies. In another study, mice were vaccinated with both DNA plasmids and alphavirus replicons expressing Rift Valley fever virus (RVFV) glycoprotein Gn fused to the C3d complement protein [54]. The immunization generated neutralizing antibodies and provided protection against challenges with RVFV, which suggested that plasmid DNA and alphavirus replicon approaches, as well as the combined DNA prime/replicon boost strategy show great promise for valid RVFV vaccine development. The combined approach included plasmid vaccinations at Weeks 0 and 3 followed by a replicon boost at Week 6.

In a combined vaccine approach, SFV DNA vectors and recombinant adenovirus expressing the classical swine fever virus (CSFV) E2 glycoprotein elicited higher titers of neutralizing antibodies in pigs [26]. After challenges with the virulent CSFV Shimen strain, no symptoms of viremia were observed, for the combined vaccine, whereas vaccination with adenovirus alone resulted in viremia in one pig of five. Furthermore, sequential immunization with SIN and VEE replicon particles expressing the type 1 HIV gp140 envelope (Env) and trimeric Env protein in MF59 adjuvant provided partial protection in macaques against intravenous challenges with high doses of simian-human immunodeficiency virus (SHIV) [61]. The administration could be further extended to intramuscular and mucosal delivery [62]. Different degrees of protection were observed against challenges with SHIV after mucosal administration. In contrast, intramuscular vaccination rendered macaques to be completely resistant to SHIV. In cotton rats, SIN DNA vectors carrying the hemagglutinin (pMSIN-H) and fusion proteins (pMSINH-FdU) elicited neutralizing antibodies, mucosal and systemic antibody-secreting cells, memory B-cells and IFNɤ secreting T-cells [47]. Priming with pMSIN-H provided 100% protection against challenges with pulmonary measles. However, pMSINH-FdU priming was observed only after a boost with live measles virus vaccine. In another study, chimeric VEE/SIN replicon particles were applied for the expression of measles virus hemagglutinin (H) and fusion (F) proteins, which elicited high-titer neutralizing antibody and IFNɤ-producing T-cells in macaques after intradermal vaccination [48]. Protection from rash and viremia was obtained after challenges with wild-type measles virus 12–17 months after vaccination. Alphaviruses have also been subjected to the development of smallpox vaccines by the introduction of A33R, B5R, A27L and L1R genes into VEE particles [59]. Vaccinated mice showed protective immunity. Furthermore, vaccination of macaques elicited strong antibody responses and was capable of neutralizing and inhibiting the spread of vaccinia and monkey pox viruses. SIN-based DNA vaccines have been developed against rabies [51]. In comparison to a conventional rabies DNA vaccine, the SIN DNA vaccine induced better humoral and cell-mediated immune responses in immunized mice and showed complete protection against challenge with the CVS rabies strain.

Recently, novel hepatitis C virus (HCV) vaccine candidates were developed by expressing all or a part of the HCV non-structural proteins (nsPs) from an SFV vector [32]. An insert as large as 6.1 kb allowed the expression of all nsPs leading to a strong and long-lasting NS3-specific CD8^+^ T-cell response. The level of T-cell response was similar to that observed for the expression of only NS3/4A. Immunization demonstrated significant growth delay of HCV-expressing EL4 tumors in a mouse model. In another study, glycoproteins for either Sudan virus (SUDV) or Ebola virus were expressed from VEE replicons and evaluated in vaccinated nonhuman primates [58]. A single intramuscular injection with VEE particles expressing SUDV GP provided complete protection against challenges with SUDV in cynomolgus macaques. However, VEE-SUDV GP vaccinated primates were not fully protected against back challenges with Ebola virus. On the other hand, co-injection of VEE particles expressing SUFV GP and Ebola virus GP showed protection against both virus types.

Recently, VEE replicon particles generated in Vero cells were used to express the E2 glycoprotein of bovine viral diarrhea virus (BVDV) [23]. Vaccination of BVDV free calves with 1 × 10^6^ IU and 1 × 10^7^ IU, respectively, resulted in neutralizing antibody titers, which were able to cross-neutralize both type 1 and type 2 BVDV genotypes after booster immunizations. Vaccination with the higher dose significantly reduced the viral-based leukopenia and showed some protection from clinical disease. Similarly, VEE replicon particles were engineered to express Dengue virus E antigen in two configurations as subviral particles (prME) and soluble dimers (E85) [27]. Immunization of macaques resulted in the rapid production of neutralizing antibodies and demonstrated protection against challenges with Dengue virus. Moreover, the tetravalent E85 VEE replicon particle vaccine induced a protective response to all four Dengue virus serotypes when two immunizations were administered six weeks apart.

A novel approach has been to combine alphavirus replicons with pseudotyped baculovirus [63]. In this context, pseudotyped baculovirus vectors containing the hybrid CM promoter and SFV replicon were used for the expression of GP5 and M proteins of porcine reproductive and respiratory syndrome virus (PRRSV) and compared to a pseudotyped baculovirus vector carrying only a CMV promoter. Immunization with the hybrid CMV/SFV showed the induction of strong GP5-specific antibodies, and in general, the Th1-dominant immune response was stronger than the one elicited by the CMV promoter alone.

Veterinary medicine has also gained from alphavirus vaccine development. In this context, salmonid alphavirus (SAV) replicons were applied for the expression of infectious salmon anemia virus (ISAV) hemagglutinin-esterase (HE) [40]. Intramuscular administration of SAV replicons provided protection against challenges with ISAV in Atlantic salmon. In contrast, intraperitoneal injection was not successful [64]. In another study, DNA vaccines based on the SAV E1 and E2 spike proteins were compared to whole virus vaccine in Atlantic salmon [65]. The whole virus vaccine showed superior immunogenicity to the DNA vaccine; the latter provided only marginal reduction in viral replication, and the protection against SAV challenges was no different from controls.

## 3. Non-Viral Targets

Alphavirus-based vaccine development has also been addressed for a number of other infectious pathogens (Table 2). For instance, SFV vectors were employed for the expression of the *Plasmodium falciparum* Pf332 antigen, which elicited immunological memory in vaccinated mice [66]. Moreover, vaccination of mice with SIN plasmid DNA vectors carrying the *Mycobacterium tuberculosis* 85A antigen (Ag85A) provided strong immunity and resulted in long-term protection against *M. tuberculosis* challenges [67]. In another approach, using SFV DNA replicons, the botulinum neurotoxin A Hc (BoNTA-Hc) gene provided both antibody and lymphoproliferative responses in vaccinated BALB/c mice [68]. The immunogenicity was enhanced when granulocyte-macrophage colony-stimulating factor (GM-CSF) was co-expressed as an adjuvant. Additionally, replication-deficient SFV particles were used for the expression of the *Brucella abortus* translation initiation factor 3 (IF3) [69]. Immunization of BALB/c mice demonstrated significant levels of protection against challenges with the virulent *B. abortus* strain 2308. Similarly, protective antigen (PA) for *Bacillus anthracis* were expressed from SIN vectors, resulting in specific and neutralizing antibodies in Swiss Webster mice and offered some protection against challenges with lethal doses of the pathogenic bacteria [70].

**Table 2:** Alphavirus-based vaccine development for non-viral infectious agents.

Agent	Target	Vector/Delivery	Immunization	Response	Reference
*B. anthracis*	PA	SIN/Particles	Mouse	*B. anthracis* protection	[70]
*B. abortus*	IF3	SFV/Particles	Mouse	*Brucella* protection	[69]
*C. botulinum*	BoNTA-Hc	SFV/DNA	Mouse	Abs, lymphoproliferative response	[68]
Malaria	CS	SIN/Particles	Mouse	Malaria protection	[71]
*M. tuberculosis*	Ag85A	SIN/DNA	Mouse	Protection	[67]
*P. falciparum*	Ag Pf332	SFV/Particles-RNA	Mouse	Immunological memory	[66]
Prion	NP	SFV/Particles	Mouse	Monoclonal Abs	[72]
Staphylococcus	enterotoxin B	VEE/Particles	Mouse	Protection	[73]

Abbreviations: Abs, antibodies; SFV, Semliki Forest virus; SIN, Sindbis virus; VEE, Venezuelan equine encephalitis virus.

## 4. Tumor Vaccine Approaches

Alphaviruses have found frequent applications in the area of tumor vaccine development (Table 3). In this context, naked RNA, replication-deficient particles and DNA layered vectors have been employed as delivery vehicles. For instance, mice have been subjected to immunization with naked SFV RNA replicons carrying the LacZ gene [74]. Interestingly, a single injection of only 1 μg of SFV‑LacZ RNA presented complete tumor protection. Furthermore, when tumors were administered two days prior to the immunization, the survival was extended by 10–20 days. Among DNA-based tumor vaccine approaches, SIN vectors expressing mouse and human tyrosine-related protein-1 (TRP-1) were evaluated in a B16 mouse melanoma model [75]. Intramuscular injection was capable of breaking immune tolerance and provided protection against melanoma when mice were vaccinated five days prior to cancer challenge. In another study, alphavirus replicon-based expression of melanoma differentiation antigen (MDA) tyrosine demonstrated the inhibition of the growth of B16 transplantable melanoma [76]. In this context, the vaccine encoding tyrosine related protein 2 (TRP-2) relied on a novel immune mechanism, which required the activation of both IgG and CD8^+^ cell effector responses.

**Table 3:** Alphavirus-based vaccine development for cancer targets.

Target	Gene	Vector/Delivery	Immunization	Response	Reference
Brain tumor	IL-12	SFV/Particles	Mouse	Immunogenicity	[77]
	Endostatin	SFV/Particles	Mouse	Inhibited tumor growth	[78]
	LacZ	SFV/Particles	Mouse	Tumor protection	[79]
	gp100, IL-18	SIN/DNA	Mouse	Tumor protection	[80]
	HER2/neu	SIN/DNA	Mouse	Tumor protection	[81]
	HER2/neu	SIN/DNA	Mouse	Tumor protection	[82]
	HER2/neu	SIN/DNA, paclitaxel	Mouse	Tumor regression	[83]
	Neu	VEE/Particles	Rat	Anti-tumor immunity	[84]
	Neu	VEE /Particles, DCs	Mouse	Tumor regression	[85]
Cervical cancer	HPVE6-E7	SFV/Particles	Mouse	Tumor protection	[86]
	HPVE6-E7	SFV/Particles	Mouse	Tumor regression	[87]
	HPV-CRT	SIN/Particles	Mouse	Tumor protection	[88]
	HPVE7	VEE/Particles	Mouse	Tumor protection	[89]
	HPVE6E7+IL12	SFV/Particles	Mouse	Anti-tumor activity	[90]
	HPVE7-VP22	SIN/Particles	Mouse	CD8^+^ T-cell response	[91]
Colon cancer	SFV vector	SFV/Particles	Mouse	Anti-tumor effect	[92]
Endothelial	VEGFR-2	SFV/Particles	Mouse	Antibody response	[93]
Glioma	B16, 203	SFV/Particles	Mouse	Tumor protection	[94]
Kidney cancer	IL-12	SFV/Encapsulated particles	Human Phase I	5-fold IL-12 expression	[95]
Melanoma	MDA/trp-2	VEE/Particles	Mouse	Therapeutic effect	[76]
	IL-12	SFV/Particles	Mouse	Tumor eradication	[96]
	IL-12	SFV/Encapsulated particles	Human Phase I	5-fold IL-12 expression	[95]
	MUC18/MCAM	SIN/DNA	Mouse	Tumor protection	[97]
Metastatic	CEA	VEE/Particles	Human Phase I	CEA Abs, extended survival	[98]
	PSMA	VEE/Particles	Human Phase I	PSMA Abs	[99]
Prostate cancer	PSMA	VEE/Particles	Mouse	Tumor response	[100]
	STEAP	VEE/DNA	Mouse	Anti-tumor response	[101]
	PSCA	VEE/Particles	Mouse	Tumor protection	[102]
Tumor	β-galactosidase	SFV/RNA	Mouse	Tumor protection	[74]
	IL-12	SFV/Particles	Mouse	Tumor protection	[103]
Tumor antigen	MHC class II	SFV/Particles-DNA	Mouse	Immunogenicity	[104]
	P815	SFV/Particles	Mouse	CTL, tumor protection	[105]
	trp-1	SIN/DNA	Mouse	Antitumor activity	[91]

Abbreviations: CEA, carcinoembryonic antigen; CRT, calreticulin; CTL, cytotoxic T-lymphocyte activity; DCs, dendritic cells; HPV, human papillomavirus; IL, interleukin; MCAM, melanoma cell adhesion molecule; MDA, melanoma differentiation antigen; MHC, major histocompatibility complex; PSCA, prostate stem cell antigen; PSMA, prostate-specific membrane antigen; SFV, Semliki Forest virus; SIN, Sindbis virus; STEAP, six-transmembrane epithelial antigen of the prostate; trp, tyrosine-related protein; VEE, Venezuelan equine encephalitis virus.

Furthermore, vaccination with recombinant particles expressing the P1A gene [105] and the human papilloma virus (HPV) E7 gene [89] from SFV and VEE vectors, respectively, provided protection against further tumor development in mice. Attempts have also been made to improve the efficacy of SFV-based HPV vaccines by supplying SFV-based IL-12 expression in mice [90]. At low doses, IL-12 stimulated antigen-specific CTL responses and enhanced anti-tumor responses after SFV-based HPV16-E6E7 immunization. Subsequent increases in dosage, however, neither improved the immune responses, nor tumor regression. SIN DNA vectors have been employed for the expression of the murine melanoma cell adhesion molecule (MCAM/MUC18) as a vaccine against murine melanoma, which resulted in the induction of humoral and CD8^+^ T-cell immune responses against melanoma [97].

In the context of breast cancer, a DNA-based SIN vector expressing the neu gene was applied for intramuscular vaccination of mice 14 days prior to the injection of cancer cells overexpressing neu [81]. The immunization provided strong protection against tumor development. The incidence of lung metastasis from mammary fat pad tumors was reduced. Moreover, the number of lung metastases from intravenous injection of neu overexpressing cells decreased. Additionally, intradermal vaccination provided tumor protection applying 80% less plasmid than required for conventional DNA vectors. Further confirmation of successful cancer vaccination was obtained from the administration of SIN vectors expressing neu (pSINCP/neu) in a murine breast tumor model [82]. However, in this case, the prophylactic vaccine only showed efficacy when administered prior to the tumor challenge. Another approach was comprised of combining alphavirus-based delivery with the chemical anticancer agent, doxorubicin [83]. When pSINCP/neu DNA and VEE/neu particles were administered after injection of 5 mg/kg of doxorubicin, the tumor progression was significantly delayed. This phenomenon did not occur for doxorubicin alone. Similarly, a combination therapy with paclitaxel (25 mg/kg) and pSINCP/neu was ineffective. Moreover, VEE-neu particles were subcutaneously administered in a rat mammary tumor model, which resulted in the elimination of 36% of pre-existing aggressive mammary tumors [84]. The combination of dendritic cell (DC)-based cancer immunotherapy with VEE-neu particle administration induced both cellular and humoral immunity against neu in transgenic human breast tumor-bearing mice [85]. Moreover, this treatment resulted in the significant inhibition of tumor growth. Similarly, both tumor growth and pulmonary metastatic spread were significantly inhibited when mice with pre‑existing tumors were subjected to five immunizations with SFV10-E VLP expressing the vascular endothelial growth factor receptor 2 (VEGFR-2) [93]. Furthermore, co-immunization with SFV particles encoding VEGFR-2 and IL-4 generated significant tumor regression in mice.

Lung cancer has also been targeted by combined therapy with SFV-IL-12 particles and anti-CD137 monoclonal antibodies [96]. Syngeneic TC-1 lung carcinoma was inhibited after intratumoral SFV-IL-12 administration and co-stimulation with anti-CD137 mAbs. In the context of colon cancer vaccines, a SIN-based DNA vector carrying the LacZ gene was compared to conventional plasmid DNA vectors in mice with CT26.CL25 tumors [106]. Intramuscular immunization elicited immune responses at doses 100- to 1,000-fold lower for the SIN DNA replicon vector compared to the conventional CMV‑promoter-based DNA-LacZ vector. Similarly, SFV particles providing VEGFR-2 expressing in vaccinated mice inhibited CT26 colon carcinoma growth [93]. Closer analysis of microvessel density demonstrated that a significant inhibition of tumor angiogenesis occurred. Additionally, when mice were co-immunized with SFV-VEGFR-2 and SFV-IL-4 particles, their survival rate was significantly enhanced. Furthermore, oncolytic SFV vectors have been used for immune stimulation in a CT26 colon tumor model [92]. Intratumoral injections led to an immediate and intense inflammatory reaction and a significant improvement in survival rates. SFV particles expressing HPV16 E6 and E7 have been subjected to prophylactic vaccine development in a murine TC-1 model for cervical cancer [107]. Pre‑immunization with a low dose (10^4^ particles) resulted in an HPV-specific CTL response in 50% of mice, whereas a higher dose (10^6^ particles) elicited CTL responses in all animals. Furthermore, at a dose of 5 × 10^6^ particles, 40% of mice were protected from tumor challenges. In another study, the SFV-enhE6,7 particle vaccine showed its potential after intravenous and intramuscular delivery, where exponentially growing tumors completely resolved [87]. Similarly, SIN virus RNA replicons expressing HPV E7 were evaluated in a TC-1 mouse model [108]. The humoral and cellular immune responses were poor, and no tumor protection was obtained; but, when the HPV E7 gene was fused to the secretory Sig protein and lysosome-associated membrane protein 1 (LAMP-1), enhanced E7‑specific CD4^+^ helper T-cell and CD8^+^ cytotoxic T-cell activity was observed. Moreover, strong *in vivo* anti-tumor activity was induced. In another study, SIN virus particles expressing both HPV E7 and calreticulin (CRT), an endoplasmic reticulum Ca^2+^ binding transporter, were tested as prophylactic vaccines [88]. Vaccinations generated antigen-specific immune responses, an anti‑angiogenic effect and a strong anti-tumor activity. Furthermore, intramuscular immunization one week prior to challenge with TC-1 carcinoma cells provided protection to all treated mice. Also VEE particles expressing HPV E7 were subcutaneously injected in mice two weeks prior to cancer cell inoculation, which prevented tumor formation [89]. Furthermore, vaccination induced long-term memory, as protection was observed for challenges three months after the immunization. The therapeutic efficacy was only 67% of treated tumor-bearing mice. However, co-expression of HPV E6 and E7 from the same vector significantly enhanced the therapeutic effect [109,110].

In a prostate tumor model, VEE particles expressing human prostate-specific membrane antigen (PSMA) showed strong cellular and humoral immunity after subcutaneous administration [100]. Furthermore, VEE particles have been employed for the expression of the predominantly prostate tissue-specific six transmembrane epithelial antigen of the prostate (STEAP) [101]. Pre‑immunization with VEE-STEAP particles induced a specific immune response and significantly prolonged the overall survival of mice bearing TRAMPC-2 tumors. When TRAMP mice were prophylactically immunized with a prostate stem cell antigen (PSCA) DNA plasmid followed by VEE‑PSCA particle administration, a specific immune response and anti-tumor protection were observed in 90% of vaccinated animals [102]. Several vaccine studies have targeted brain tumors. For instance, SFV particles expressing endostatin showed a significant reduction of intratumoral vascularization after intratumoral delivery [78]. In another approach, bone-marrow isolated dendritic cells (DCs) were transduced with SFV vectors carrying cytokine genes of specific cDNAs from melanoma and glioma cells [79]. Pre-vaccination with DCs transduced with SFV-based B16 and 203 glioma cDNAs, respectively, resulted in tumor challenge protection and the prolonged survival of tumor-bearing mice. The combination of DCs transduced with SFV-IL-12 particles and systemically administered IL-18 also provided increased survival rates [111]. Moreover, the expression of human melanoma-associated antigen gp100 and IL-18 from a SIN virus DNA vector induced specific anti-tumor CTL responses and provided anti-tumor protection [80]. Vaccination prevented B16-hgp100 tumor formation and demonstrated significant prolongation of survival in mice with established B16-hgp100 tumors.

## 5. Vaccines against Alphaviruses

As alphaviruses have been suggested to be responsible for epidemics in various parts of the world, it has also become important to develop vaccines against alphaviruses themselves (Table 4). In this context, protection against airborne virus was observed in BALB/c mice vaccinated with an attenuated VEE strain [112]. In another study, improved protection against VEE challenges was achieved by using a live attenuated V3526 VEE vaccine [113]. Similarly, when a chimeric Eastern equine encephalitis (EEE) and Western equine encephalitis (WEE) virus were applied for the vaccination of C57BL/6 mice, complete protection was observed against lethal challenges with a virulent Eastern equine encephalitis (EEE) virus strain [114].

**Table 4:** Alphavirus-based vaccine development against alphaviruses.

Virus	Gene	Vector Delivery	Immunization	Response	Reference
CHIK	TSI-GSD-218	CHIK infection	Human Phase II	Neutralizing Abs	[115]
CHIK	Glycoprotein	CHIK infection	Macaques	Neutralizing Abs	[116]
CHIK	IRES	CHIK infection	Vero cells	Mosquito resistance	[117]
CHIK	C, E1 VLPs	CHIK infection	Primates	CHIK protection	[118]
CHIK	nsP3, E1 siRNA	CHIK infection	Vero cells	Reduced CHIK titer	[119]
CHIK	miRNAs	CHIK infection	Mouse	Reduced CHIK replication	[120]
EEE	EEE/WEE	EEE infection	Mouse	EEE protection	[114]
VEE	VEE att	VEE infection	Mouse	VEE protection	[112]
VEE	VEE V3526	VEE infection	Mouse	VEE protection	[113]
VEE	VEE TC-83	VEE infection	Mouse	VEE protection	[121]
VEE	26S	VEE infection	Macaques	VEE protection	[122]
VEE	CHIK genes	Chimeric VEE-CHIK	Mosquito	Reduced infectivity	[123]
VEE	RdRp miRNA	VEE infection	BHK cells	Inhibition of VEE replication	[124]
WNV	WNV att	WNV Nanopatch	Mouse	Abs	[125]

Abbreviations: Abs, antibodies; att, attenuated; C, capsid; CHIK, Chikungunya virus; EEE, Eastern equine encephalitis virus; miRNA, micro-RNA; RdRp, RNA-dependent RNA polymerase; SFV, Semliki Forest virus; SIN, Sindbis virus; siRNA, short interfering RNA; VEE, Venezuelan equine encephalitis virus; VLPs, virus-like particles; WNV, West Nile virus.

In attempts to design attenuated alphaviruses for vaccine development, the mechanisms of replication and virus-host interaction have been investigated. In this context, mosquito transmission of Chikungunya (CHIK) virus has been prevented by making their replication dependent on internal ribosome entry sites (IRES) [117]. Although replication does not occur in mosquito cells, replication proceeds efficiently in Vero cells. In another approach, the non-structural genes of the attenuated VEE strain TC-83 and the naturally attenuated EEV strain, respectively, were engineered with the structural genes of CHIK [123]. The chimeric vaccine candidates presented a significantly reduced infectivity of the common urban *Aedes aegypti* and *A. albopictus* vectors for CHIK, thereby providing a low risk of virus transmission. A new CHIK virus isolated from an acutely infected human patient was used for the engineering of a synthetic DNA vaccine [116]. When this DNA vaccine was electroporated into mice, robust antigen-specific cellular and humoral responses were obtained, which provided protection against further challenges with CHIK. Furthermore, immunization of macaques demonstrated the induction of neutralizing antibodies similar to those elicited in sera from convalescent human patients. In another DNA vector approach, the VEE 26S structural genes were expressed and administered as an aerosol in nonhuman primates [122]. Vaccination resulted in no viremia in two macaques and low viremia in one animal, whereas it was high in all control animals.

In an approach to target epidermal and dermal antigen presenting cells (APCs), the Nanopatch (NP) technology has been applied for skin vaccination of West Nile virus and CHIK in mice [125]. NP, comprised of arrays of densely packed projections two orders of magnitude smaller than standard needles, provided efficient delivery of inactivated whole CHIK vaccine and a DNA-based West Nile virus vaccine. Moreover, as virus-like particles (VLPs) consisting of CHIK capsid and envelope proteins have been demonstrated to protect nonhuman primates against infection of multiple strains of CHIK, it was of benefit to screen and optimize the solution conditions for stable vaccine formulations [118]. In this context, sugar, sugar alcohols and polyanions were identified as potential stabilizers.

The relatively recent discovery of the commonly occurring phenomenon of gene silencing has also made its impact on vaccine development. In this context, small interfering RNAs (siRNAs) were engineered against two conserved regions of the nsP3 and E1 genes of CHIK [119]. A significant reduction of virus titer was observed in Vero cells after 24 h (99%) and 48 h (65%), suggesting a potential new therapeutic approach. In another approach, microRNA (miRNA)-specific target sequences for replicon particle production were introduced into alphavirus helper RNAs [120]. In the presence of miRNA-specific inhibitors, CHIK particles were efficiently generated, whereas in their absence, cellular miRNAs downregulated helper RNA replication. When mice were inoculated with replicon RNA carrying engineered miRNA sequences, cellular miRNAs prevented the replication of replicon RNA, which opens the feasibility of using miRNAs as a therapeutic approach for the inhibition of viral replication. Moreover, the VEE RNA-dependent RNA polymerase (RdRp) was targeted by artificial miRNAs in attempts to inhibit VEE replication [124]. Of five miRNAs that were evaluated in BHK cells, three showed significant inhibition of VEE replication.

## 6. Clinical Trials for Alphavirus Vaccines

Despite the large number of studies conducted in various animal models, relatively few evaluations have been subjected to humans. A clinical trial conducted with alphaviruses involved intravenous administration of liposome encapsulated SFV particles expressing IL-12 particles to melanoma and kidney carcinoma patients [95]. High transient expression of recombinant IL-12 with a five-fold increase lasted for 4–7 days. As higher doses induced a fever response in patients, the maximum tolerated dose (MTD) was restricted to 3 × 10^9^ encapsulated particles per m^2^. No liposome- or SFV-related toxicity was observed. Furthermore, the liposome-encapsulation prevented SFV particles from being recognized by the host immune system and, therefore, allowed repeated systemic administration. The phase I study clearly demonstrated the safe administration of encapsulated SFV particles.

A human phase II, randomized, double-blind, placebo-controlled, safety and immunogenicity study was conducted in 73 healthy adult volunteers on a serially passaged, plaque-purified, live CHIK vaccine [115]. A single subcutaneous injection of the CHIK vaccine was administered to 59 volunteers, and 14 individuals received placebo. There were no adverse events, except for transient arthralgia in five individuals receiving the CHIK vaccine. Of the 58 evaluable vaccinated individuals, 57 (98%) developed CHIK neutralizing antibodies by Day 28 and 85% still remained seropositive one year later. None of the individuals who received placebo were seropositive. In another vaccine-related alphavirus phase I randomized, double-blind clinical trial for cytomegalovirus (CMV), a two-component vaccine expressing CMV gB or pp65/1E1 fusion protein was administered intramuscularly or subcutaneously in CMV seronegative adult volunteers [25]. The vaccination showed only mild to moderate local reactions and no clinically important changes. It induced neutralizing antibody and multifunctional T-cell responses against CMV antigens.

In another clinical trial, alphavirus particles expressing the carcinoembryonic antigen (CEA) were repeatedly administered to patients with metastatic cancer [98]. CEA-specific antibodies were able to mediate antibody-dependent cellular cytotoxicity against tumor cells from human colorectal cancer metastases. Furthermore, patients with CEA-specific antibodies encouragingly showed extended overall survival. Similarly, VEE replicons expressing the prostate-specific membrane antigen (PSMA) were applied for a human trial of patients with metastatic cancers at five doses of 0.9 × 10^7^ IU or 3.6 × 10^7^ IU [99]. At the lower dose, no PSMA-specific cellular response was obtained, but a weak PSMA-specific signal was detected by ELISA. Disappointingly, the higher dose showed no PMSA‑specific response. The trial demonstrated that while there was neither clinical benefit nor robust immune responses, there were no toxicities associated with the immunization, and the VEE-PMSA particles were well tolerated. However, as neutralizing antibodies were elicited, the dosing might be suboptimal, requiring some further optimization.

## 7. Conclusions

A number of vaccine development studies have been conducted using mainly the three most commonly applied alphavirus vectors, SFV, SIN and VEE. To demonstrate the variety of approaches available, replicon particles, naked RNA and layered DNA vectors have been employed. Each approach has commonly generated responses detected by cellular or humoral responses. In the case of vaccine development against lethal viruses (Table 1), non-viral infectious targets (Table 2) and tumors (Table 3) immunization has provided long-term protection against challenges with the disease-causing agents. Moreover, due to the pathogenicity of several alphaviruses, they are themselves credible targets for vaccine development. Therefore, a number of studies, particularly for VEE and CHIK (Table 4), have provided protection against challenges with virulent alphavirus strains.

The potential of alphaviruses causing global epidemics has placed additional concern on the needs of addressing biosafety issues. Generally, the strains used for vaccine development are attenuated. Furthermore, in the case using alphavirus particles, second generation helper vectors [126] or split helper systems [8] have been applied to prevent any production of wild-type-like replication-proficient particles through non-homologous recombination. Only recently alphavirus-based vaccines have been subjected to clinical trials. In this context, SFV vectors have been used for delivery of immunostimulatory genes. Furthermore, immunizations for the treatment of CMV and vaccinations against prostate and metastatic cancers have been conducted with alphavirus vectors and particles. The strength of applying alphaviruses is the generation of rapid transgene expression and the transient nature of expression. However, the full potential of alphavirus-based vaccines has not been explored yet. In the future, it is anticipated that additional, positive observations, particularly in the form of providing protection against challenges with lethal pathogens and tumors, will attract the enhanced application of alphaviruses for vaccine development.
